# Minimally invasive anatomic reconstruction of the anterolateral ligament with ipsilateral gracilis tendon: a kinematic in‐vitro study

**DOI:** 10.1186/s40634-022-00543-2

**Published:** 2022-10-22

**Authors:** Giulio Maria Marcheggiani Muccioli, Vito Gaetano Rinaldi, Marcello Zappia, Giada Lullini, Simone Bignozzi, Stefano Zaffagnini, Giovanni Felice Trinchese

**Affiliations:** ^1^ II Clinica Ortopedica e Traumatologica IRCCS Istituto Ortopedico Rizzoli Bologna Italy; ^2^ DIBINEM University of Bologna via di Barbiano, 1/10 – c/o Lab Biomeccanica ed Innovazione Tecnologica 40136 Bologna Italy; ^3^ Department of Medicine and Health Science University of Molise Campobasso Italy; ^4^ Department of Radiology Varelli Insitute Naples Italy; ^5^ U.O.C. Medicina Riabilitativa e Neuroriabilitazione IRCCS Istituto delle Scienze Neurologiche di Bologna Bologna Italy; ^6^ Orthokey Srl Florence Italy; ^7^ Department of Orthopaedics and Traumatology Villa Malta Hospital Sarno SA Italy

**Keywords:** Anterolateral ligament, Knee joint, Anatomical reconstruction, In‐vitro study, Laxity, Kinematics

## Abstract

**Purpose:**

The anterolateral ligament (ALL) has been defined as a key stabilizer of internal tibial rotation at 35° or more of knee flexion, with a minimal primary or secondary stabilizing role in the AP direction. This study aimed to demonstrate that anatomical reconstruction of the ALL confers rotational stability equal to that of the uninjured knee. Hypothesis: anteroposterior (AP) and rotatory laxity will significantly vary after ALL tenotomy and ALL reconstruction with the author’s previously described technique.

**Methods:**

After ultrasound (US) ALL identification, different kinematic measurements were performed with an image‐less Computer‐Assisted Navigation System with dedicated software for Laxity Analysis in 5 knee specimens. Anteroposterior (AP) translations and varus/valgus (VV) and Internal‐External (IE) rotations were evaluated by two trained orthopedic surgeons before ALL section, after ALL section, and after ALL anatomical reconstruction with doubled ipsilateral autologous gracilis tendon.

**Results:**

ALL resection significantly increased laxity in IE rotations with knee 90° flexed (IE90) and AP translation with tibia internally rotated and the knee 30° flexed (APlat) (*p* < 0.05). ALL reconstruction significantly reduced laxity in IE90 and APlat (*p* < 0.05) and reduced VV rotations at 30° of flexion (VV30) (*p* < 0.05).

There were no statistically significant elongation differences between native ALL and reconstructed ALL (graft) during laxity tests. The inter‐operator repeatability of the tests was excellent for each measurement.

**Conclusions:**

ALL acted as an important internal tibial rotation restrain at 90° and a significant (secondary) AP stabilizer at 30° of knee flexion. The presented ALL reconstruction technique significantly restored the increase of knee laxity produced by the ALL section.

**Scientific level:**

Case‐Controlled Laboratory Study, Level III.

## Introduction

The anterior lateral complex (ALC) of the knee consists of the anterolateral ligament (ALL), the Kaplan fibers, and the iliotibial band [12, 27].

Untreated anterolateral injuries, in the presence of an Anterior Cruciate Ligament (ACL) deficiency, result in abnormal knee laxity when only treated with intra‐articular ACL reconstruction [22, 35]. In addition, recent studies have pointed out that ALL reconstruction associated with ACL reconstruction improves knee stability and patients satisfaction [3, 21].

As a matter of fact, the ALL has been reported to be a key stabilizer of internal tibial rotation at 35°or more of knee flexion, with a minimal primary or secondary stabilizing role in the AP direction [20, 30], showing Magnetic Resonance Imaging (MRI) abnormalities from 40% to 88% in ACL‐injured knee according to Helito et al. [19] and Ferretti et al. [14], respectively. Due to its importance in knee rotatory stabilization, ALL reconstruction became a crucial topic and source of controversy.

Many authors have recently debated the best imaging method to visualize the ALL and its potential lesions [4, 15, 25, 33].

Several studies have evaluated the ability of MRI to identify the ALL in the injured knee. In particular, Claes et al. reported that MRI could identify the ALL in only 76% of selected patients [10]. In comparison, Helito et al. reported a percentage of only 71.7% identification of the ALL with a 1.5 T MRI unit [18].

In recent years, assessment of ALL by ultrasound has been gaining popularity with excellent results [4, 5, 7, 25].

In particular, in recent articles published in the literature Cavaignac et al. [6, 7], and Cianca et al. [9] reported that via ultrasonography it is possible to identify ALL with absolute accuracy in 100% of cases.

Moreover, Oshima et al. [29] reported that almost all the ALL segments could be identified through ultrasonography, making it a practical examination for diagnosing ALL injuries.

This study aimed to study the effect of anatomical reconstruction of the ALL to confer rotational stability equal to that of the uninjured knee.

All the ALL reconstructions were performed using an innovative, minimally invasive anatomic technique using the ipsilateral autologous gracilis tendon, previously described by the Authors [39].

We hypothesized that ALL reconstruction with the described technique will significantly reduce the anteroposterior and rotatory knee laxity.

## Materials and methods

### Study design

Five fresh‐frozen knee specimens were provided for this study by the ICLO Teaching and Research Center (Arezzo, Italy), and all the specimens were thawed at room temperature for 24 hours before preparation.

The study was conducted following approval from the Ethics Committee at our institution.

Each knee underwent an initial ultrasound (US) analysis performed by the same expert trained musculoskeletal radiologist to identify the ALL, followed by kinematic analysis of the knee.

First, the knee laxity was evaluated using an image‐less Computer‐Assisted Navigation System. Once the anatomical dissection was carried out, the kinematic analysis was performed after the ALL section and finally with anatomically reconstructed ALL.

### Ultrasound ALL identification

Before the anatomical dissection, an ultrasound (US) examination was performed on each knee by a musculoskeletal radiologist with more than 15 years of professional background (M.Z.). Sonography examinations were performed using an HM70 machine (Samsung Healthcare, Seoul, Republic of Korea) using a linear probe 3‐16Mhz. All US examinations were performed to detect the anatomical landmarks (Gerdy’s tubercle, Lateral collateral ligament (LCL), iliotibial band, popliteus tendon, and ALL) with the knee flexed at 10° and 10° of internal rotation to give some tension to the ALL. Thus, the ALL was first assessed distally by identifying Gerdy’s tubercle and looking for a ligamentous structure beginning posterior to the tubercle with directionality pointing toward the footprint of the LCL on the lateral femoral condyle, as described in literature [41].

Once the probe was positioned to obtain a correct long axis scan of the ALL, the radiologist drew its structure with a permanent marker on the skin of the knee, following the ligament course.

After performing a percutaneous dissection of the ALL at the level of the middle third using a lance‐shaped scalpel, US was repeated to ensure that the ligament had been dissected to the total thickness and that the collateral ligament and iliotibial band had not been injured.

### Computer‐assisted laxity analysis and anatomic reconstruction

After ultrasound (US) ALL identification, different kinematic measurements (see below) were evaluated by two trained orthopedic surgeons (GMMM, GFT) with an image‐less Computer‐Assisted Navigation System with dedicated software for Laxity Analysis (CAS‐LA). A navigation system (BLU‐IGS, Orthokey, Lewes, Delaware, DE, USA) equipped with dedicated software (KLEE, Orthokey, Florence, Italy), commonly used for intra‐operative kinematic analysis, was here adopted. This methodology and software have been previously validated with reported accuracy lower than 1 mm\1° [4].

The data were processed offline using a MATLAB interface specifically developed (The Mathworks Inc., Natick, Massachusetts, MA, USA).

To quantify the ALL’s contribution to knee stability, all these measurements were repeated firstly with intact limb, then after ultrasound detection and resection of the ALL ligament, and finally after anatomic ALL reconstruction, using the previously described authors’ technique [39].

**Fig. 1:**
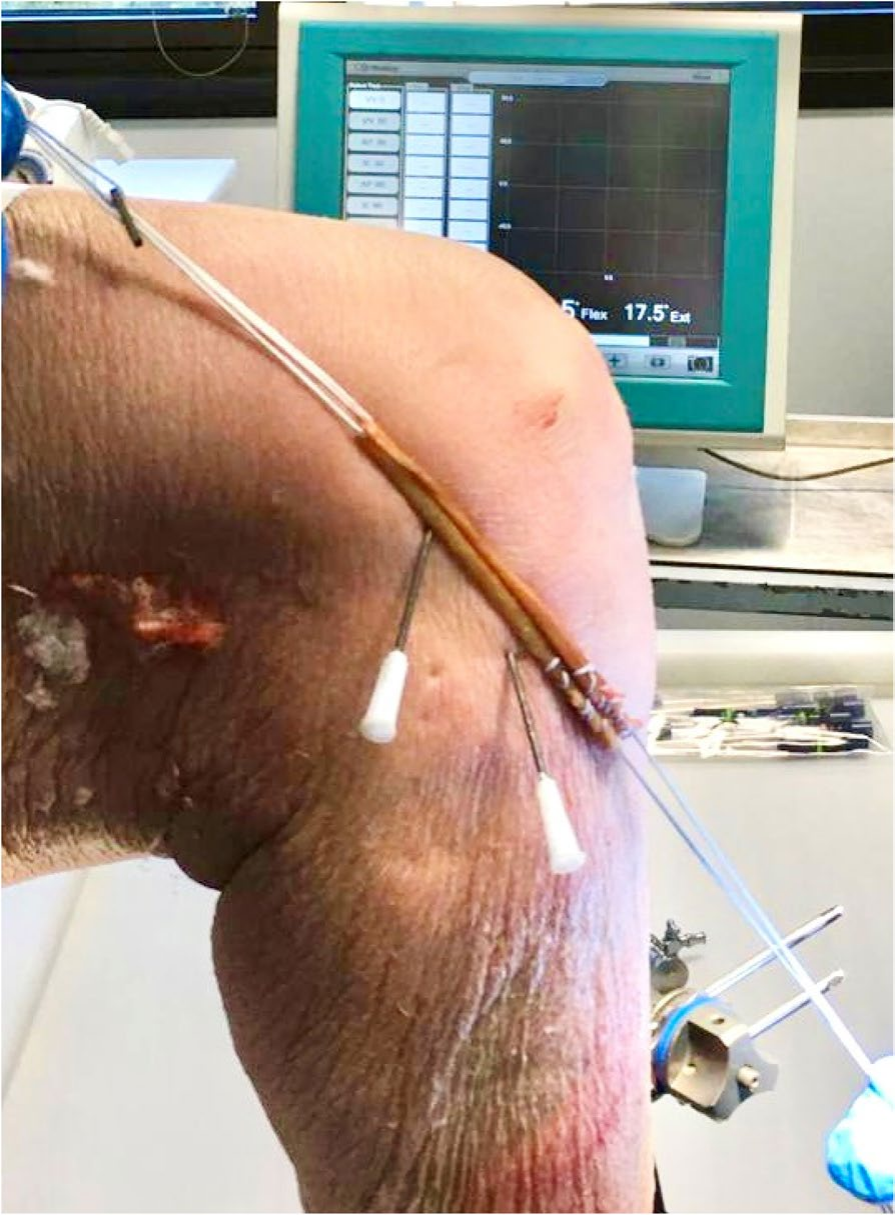
Assembled gracilis tendon and anatomic ALL landmarks marked with two needles

**Fig. 2:**
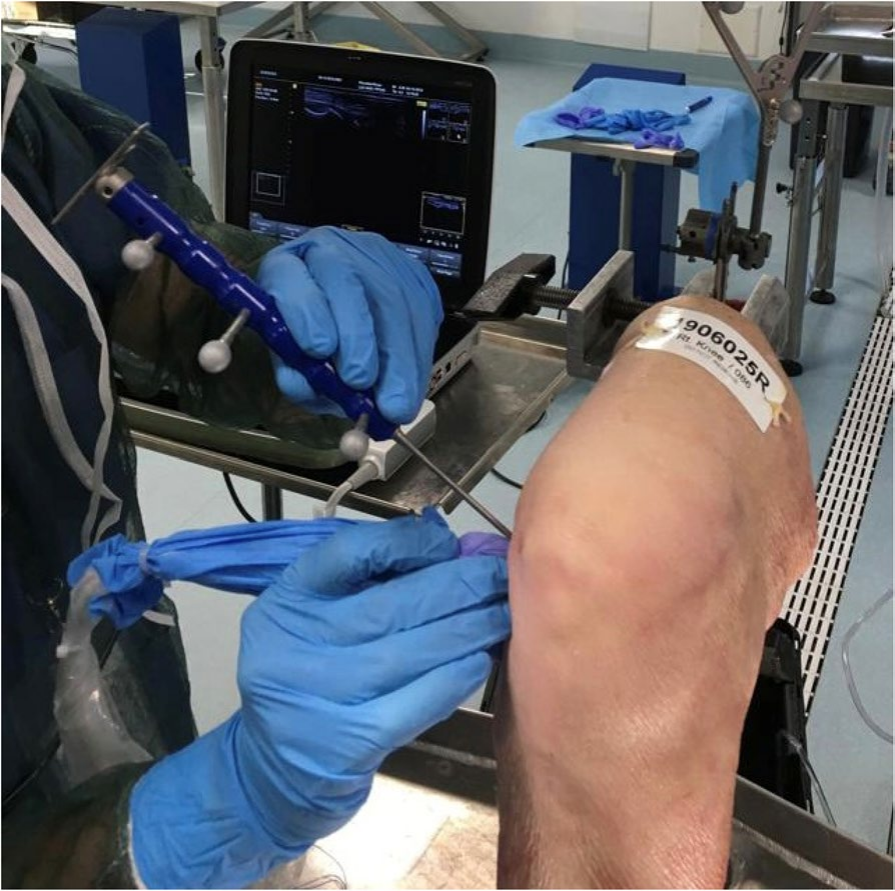
Anatomic ALL insertions assessed by ultrasonography

**Fig. 3:**
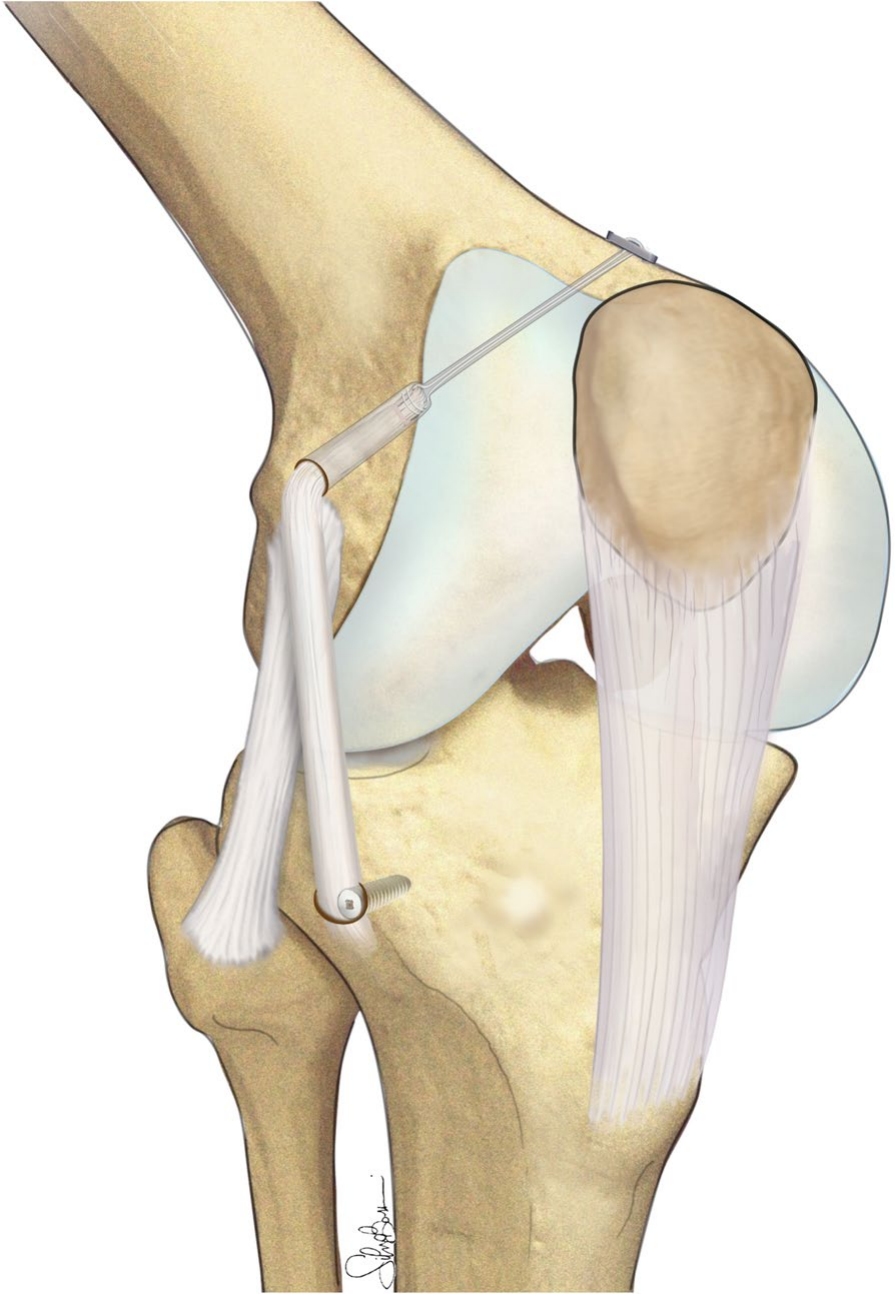
Minimally invasive ALL reconstruction with gracilis tendon. The graft is fixed at the tibial insertion with a Bio‐tenodesis screw, successively passed under the fascia lata, and locked into the femoral tunnel with an adjustable cortical fixation device

First, the gracilis tendon was harvested in the standard fashion and was prepared with non‐absorbable stitches (FiberWire N.2) and doubled to a length of at least 80 mm. Therefore, the tendon was assembled on a TightRope RT (Arthrex, Naples, FL, USA), which will fix the neo‐ligament into the femur. Second, the anatomic landmarks for the attachment and insertion of the ALL were identified. The femoral lateral epicondyle, the fibular proximal head, the Gerdy’s tubercle, the joint line, and the tibial insertion of the ALL were marked (Figs. 1 and 2). Thus, a Kirschner wire was inserted through a small cutaneous incision from the lateral to medial femoral side at the anatomic attachment of the ALL located posterior and proximal to the lateral epicondyle. A 4 mm tunnel was drilled with a cannulated drill: Consequently, a half tunnel wide enough for the duplicated gracilis (usually 5 mm) and 25 mm deep was produced at the femoral attachment. A half tunnel (20 mm long; 5 mm wide) was made at the tibial insertion of the ALL. Thereafter, the neo ligament was fixed at the tibia with a 6.25 × 15 mm Bio‐tenodesis screw (Arthrex, Naples, FL, USA) and then passed under the fascia lata with a shuttle suture. At this point, the TightRope RT (Arthrex, Naples, FL, USA) was inserted into the femoral tunnel, fixed on the femoral medial cortex, and then tensioned at 30° of knee flexion (Fig. 3).

The joints were manually tested at maximum force, measuring knee laxity with CAS‐LA during 6 different classic passive laxity tests, namely varus/valgus (VV) rotation at 0° and 30° of flexion, anterior‐posterior (AP) translation at 30° and 90° of flexion and internal/external (IE) rotation at 30° and 90° flexion. Moreover, AP translation of lateral compartment with the tibia internally rotated and the knee 30° flexed (APlat) was measured. The anterior‐posterior displacement of the tibial plateau, distinguishing the lateral from the medial compartment, was evaluated during AP30, AP90, IE30, and IE90 tests. In addition, the whole tibial rotation of the tibia was calculated during VV0, VV30, IE30, and IE90. The anterior‐posterior displacement of the tibial plateau, distinguishing the lateral from the medial compartment, was evaluated during the AP30, AP90, IE30, and IE90 tests. Full rotation of the tibia was assessed during the VV0, VV30, IE30, and IE90 tests.

Three cycles were recorded per position by each examiner.

The value given by the differences between native, dissected and reconstructed ALL test results was defined as a reduction of laxity.

Native ALL and reconstructed ALL (graft) elongation analysis was also performed.

### Statistical analysis

A nonparametric test was used to compare the three experimental phases because of the limited sample size. The Kruskal‐Wallis test was used to evaluate the mean test difference across the three phases.

All statistical analyses were performed using Analysit (Analyse‐it Software Ltd., Leeds, UK) plug‐in for Excel(Microsoft, Redmond, Washington, USA).

Power analysis was based on the repeatability of this methodology for manual testing at maximum force, as reported by Bignozzi et al. [4], and with the aim to detect at least 2 mm and 2° of difference between testing conditions. Which was considered the minimum difference that could lead to some clinical significance.

## Results

### Knee laxity analysis

ALL resection significantly increased laxity in IE90 and APlat (*p* < 0.05).

ALL reconstruction significantly reduced laxity in IE90 and APlat (*p* < 0.05) and reduced VV30 (*p* < 0.05).

**Fig. 4:**
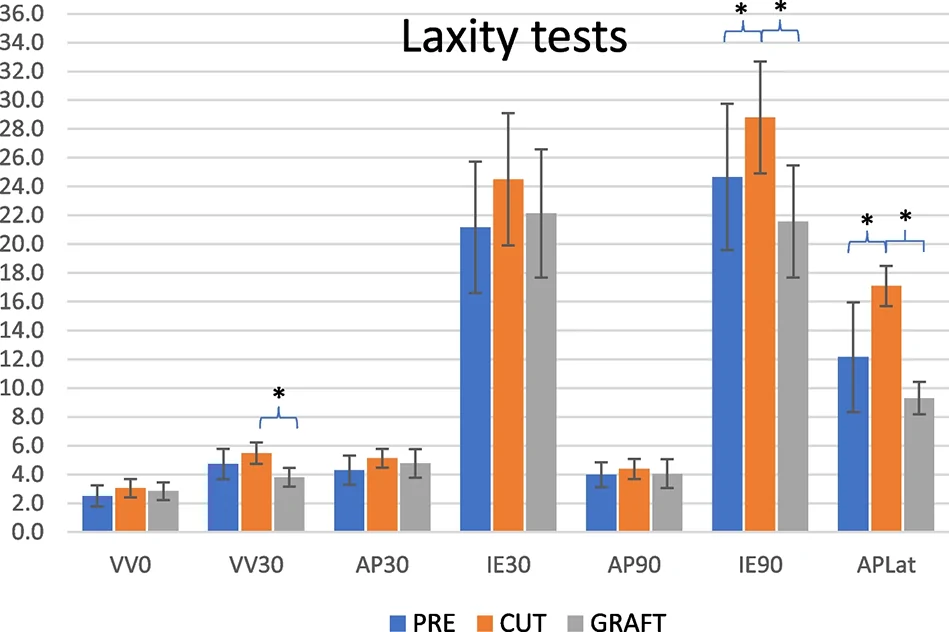
Histogram representing the mean values of laxity measured during test sessions. ALL resection significantly increased laxity in IE90 and APlat (* = *p* < .05). Nevertheless, ALL reconstruction significantly reduced laxity in IE90 and APlat (* = *p* < .05) and reduced VV30 (* = *p* < .05)

Figure 4 shows the mean values of knee laxity measured during testing.

### Native ALL and reconstructed ALL (graft) elongation analysis

**Fig. 5:**
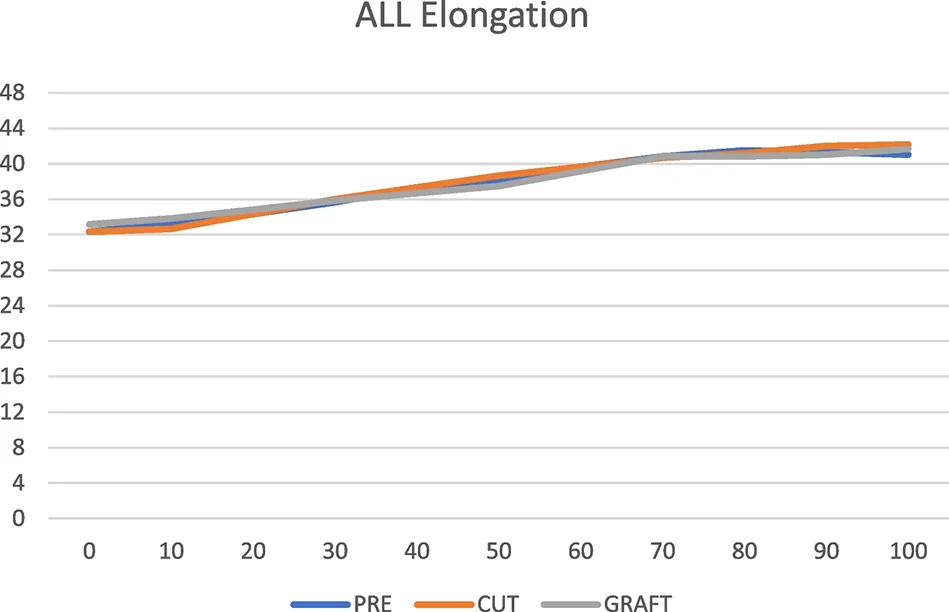
ALL elongation test shows distance modification of the ALL ligament insertion points during the various experimental phases. ALL appears to have an extension to flexion elongation length ranging from 33 mm (in extension) to 40 mm (at 100° of flexion). There were no statistically significant elongation differences between native ALL and reconstructed ALL (graft) during laxity tests

ALL appears to have an extension to flexion elongation, with a length ranging from 33 mm (in extension) to 40 mm (at 100° of flexion). There were no statistically significant elongation differences between native ALL and reconstructed ALL (graft) during laxity tests. Mean values are shown in Fig. 5.

### Repeatability analysis

**Fig. 6:**
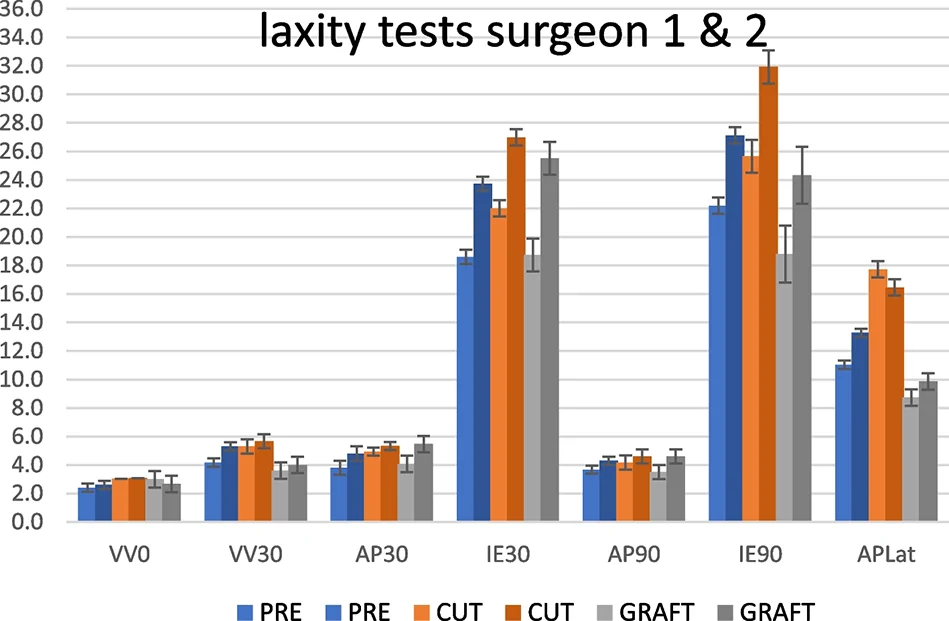
Comparative Test of laxity between the 2 surgeons. The inter‐operator repeatability of the tests was excellent for each measurement. However, differences were noted between the 2 operators, especially for the internal and external rotation tests. Nevertheless, within the same subject, the variations between pre, post, and graft were comparable within the same subject

The inter‐operator repeatability of the tests was excellent for each measurement. However, differences were noted between the 2 operators, especially for the internal and external rotation tests. Nevertheless, we can affirm that variations between pre, post, and graft within the same subject were comparable. The values of measured laxities are shown in Fig. 6.

## Discussion

The most important finding of the present study was that ALL acted as an essential internal tibial rotation restrain at 90° and as a significant (secondary) AP stabilizer at 30° of knee flexion.

The presented ALL reconstruction technique significantly restored the increase of knee laxity produced by the ALL section.

ALL resection and reconstruction showed the most significant effects on internal rotation, modifying the internal rotation at 90° of knee flexion (IE90) and the AP translation of lateral compartment with the tibia internally rotated at 30° of flexion (APlat). These previous confirmed findings from studies performing investigations on knee biomechanics reporting that the ALL has a role in controlling rotational stability [1, 8, 11, 13, 25, 30, 36]. Sonnery‐Cottet et al. [36], and Parsons et al. [30] reported that the ALL has a significant role in controlling internal rotation with a deficient ACL. Moreover, Nitri et al. [28] reported that in a combined ACL and ALL injury, the only way to restore rotatory stability was to reconstruct both structures and not just the isolated ACL.

However, not all studies in the literature are in line with those findings. Kittl et al. [24] reported only a minor contribution of the ALL on internal rotation stability with a deficient ACL. In an anatomic and biomechanical study by Rahnemai‐Azar et al. [31], the anterolateral capsule lacked any structural or biomechanical properties of a ligament, raising doubts on the existence of the ALL as a distinct structure (vs. solely a capsular thickening).

Moreover, ALL reconstruction was revealed to significantly reduce VV rotations at 30° of flexion (VV30). This could be a result of the experimental in‐vitro setup or could be due to the absence of quadriceps, hamstrings, and iliotibial band forces [17, 26].

Nonetheless, sectioning of the ALL resulted in a statistically significant increase in anterior translation, apart from internal rotation, consequently to an ACL sectioning during an early phase pivot shift [37]. Similar findings were also published in a related study [32], clearly highlighting an increase in internal rotation, consequent to ALL sectioning, using a 6‐degree‐of‐freedom robot. In addition, a recent systematic review showed that lateral extra‐articular tenodesis (LET) procedures restricted internal tibial rotation in biomechanical and clinical studies. The latter supports the anterolateral structures’ role in controlling the tibial internal rotation [34].

Regarding the High‐resolution real‐time ultrasonographic examination, we also found that, in experienced hands, it is adequate to verify the integrity of the ALL.

Previous literature concerning anatomical research has been focusing on the appearance of the ALL [14, 18, 38]: However, only a few studies were performed with US examination.

Capo et al. [5] performed an ultrasonographic evaluation on 10 fresh‐frozen cadaveric knees with a 14 MHz linear transducer: in this study, the ultrasonographic examination did not always correctly locate the origin and insertion of the ALL, with the latter often being difficultly isolated from the surrounding structures, even at dissection. The primary imaging difficulty was identifying and assessing the proximal portion of the ALL correctly; even though the distal part was reliably identified with the US on each cadaver in a similar location, it could be effectively isolated during dissection.

Literature has reported that the ALL originates posterior and proximal to the lateral epicondyle, with slight variation regarding the exact origin site, carrying a mean footprint width of 8.3 mm [15, 16, 23].

The ALL has a fan‐like blending of fibers at its femoral origin, without a distinct area of direct attachment to the femur [13]: Therefore, it is controversial whether the proximal origin of ALL is anterior to or posterior to the LCL footprint [11, 13].

In the present study, the origin of the ALL appeared to be posterior and proximal to the LCL insertion site and to the lateral epicondyle in all cadavers under US investigation, as previously demonstrated by the ALC consensus meeting [16]. However, a precise distinction concerning the two ligaments’ fiber has proved to be complicated. The ALL then runs from the region of the lateral femoral epicondyle anteroinferiorly towards the proximal tibia, obliquely across the knee, passing under the fascia lata and above the LCL, with a thickness of approximately 2 mm, and a mean width at the lateral joint line of 6.7 mm. Van derWatt et al. [40] also report that, during its course, the ALL runs deep to the ITB until its insertion and may be connected with the periphery of the body of the lateral meniscus through its meniscofemoral and meniscotibial components. Coherently, the US investigation performed in this study confirmed this anatomical description in all examined ALLs. The distal insertion site of the ALL is at the anterolateral proximal aspect of the tibia, just distal to the lateral joint margin: It is placed at a point midway between the head of the fibula and the Gerdy’s tubercle, with a mean insertion footprint width of 11.3 mm [2]. Again, the present findings confirmed previous claims.

The present study should be considered in light of the current limitations.

First, the setup did not simulate weight‐bearing activity, with muscle contraction in the physiologic direction, which has been suggested to enhance knee stability [33] because the forces of this experiment may be larger in‐vivo.

Although the forces in this experiment would likely be exceeded in vivo, the analysis compared one condition (ALL lesion) to another (ALL section); therefore, the nature of the changes is unlikely to alter, although it is acknowledged that they may be larger in‐vivo.

Second, the use of cadaveric knees with normal anatomy and a mean specimen mean age of 59 years. This limited the direct extrapolation of results to a population of patients with ALL lesions who may be younger.

Nevertheless, this study takes into consideration the knee with only ALL injury and intact anterior cruciate ligament so that we were able to evaluate the contribution of ALL to knee stability and the effectiveness of our reconstruction technique in restoring the native stability.

Further studies should investigate outcomes using alternative grafts and fixation methods.

## Conclusions

ALL acts as an important internal tibial rotation restrain at 90°of knee flexion and as an effective (secondary) AP stabilizer at 30° of knee flexion. The presented ALL reconstruction technique significantly restored the increase of knee laxity produced by the ALL section.

## Authors’ contributions

GMMM, GFT, and MZ have performed the in vitro study tests; SB, VGR, and GMMM analyzed and interpreted the data; VGR and GMMM were the major contributors to writing the manuscript, and GL and SZ substantively revised it. The author(s) read and approved the final manuscript.

## Funding

This research did not receive any specific grant from funding agencies in the public, commercial, or not‐for‐profit sectors.

## Availability of data and materials

The datasets used and analyzed during the current study are available from the corresponding author on reasonable request.

## Declarations

### Ethics approval and consent for publication

The present in‐vitro study has been promoted by Istituto Ortopedico Rizzoli, Bologna (Italy) and ICLO Teaching and Research Center (Arezzo, Italy).

### Consent for publication

All consents for publication have been collected.

### Competing interests

The authors declare that they have no competing interests.

## Publisher’s Note

Springer Nature remains neutral with regard to jurisdictional claims in published maps and institutional affiliations.

## References

[1] Amis AA Anterolateral knee biomechanics. Knee Surg Sports Traumatol Arthrosc (2017) 25(4):1015–1023 10.1007/s00167-017-4494-x PMC5420373 28299387

[2] Ariel de Lima D , Helito CP , Lacerda de Lima L , de Castro SD , Costa Cavalcante ML , Dias Leite JA Anatomy of the anterolateral ligament of the knee: a systematic review. Arthroscopy (2019) 35(2):670–681 10.1016/j.arthro.2018.09.006 30612770

[3] Ariel de Lima D , de Lima LL , de Souza NGR , de Moraes Perez RA , Sobrado MF , Guimarães TM , Helito CP Clinical outcomes of combined anterior cruciate ligament and anterolateral ligament reconstruction: a systematic review and meta‐analysis. Knee Surg Relat Res (2021) 33:33 10.1186/s43019-021-00115-1 PMC8461945 34556187

[4] Bignozzi S , Lopomo N , Martelli S , Bruni D , Marcacci M Accuracy, reliability, and repeatability of navigation Systems in Clinical Practice. Oper Tech Orthop (2008) 18:154–157 10.1053/j.oto.2008.11.001

[5] Capo J , Kaplan DJ , Fralinger DJ , Adler RS , Campbell KA , Jazrawi LM , Alaia MJ Ultrasonographic visualization and assessment of the anterolateral ligament. Knee Surg Sports Traumatol Arthrosc (2017) 25(10):3134–3139 10.1007/s00167-016-4215-x 27344549

[6] Cavaignac E , Faruch M , Wytrykowski K , Constant O , Murgier J , Berard E , Chiron P Ultrasonographic evaluation of anterolateral ligament injuries: correlation with magnetic resonance imaging and pivot‐shift testing. Arthroscopy (2017) 33(7):1384–1390 10.1016/j.arthro.2017.01.040 28343806

[7] Cavaignac E , Wytrykowski K , Reina N , Pailhé R , Murgier J , Faruch M , Chiron P Ultrasonographic identification of the anterolateral ligament of the knee. Arthroscopy (2016) 32(1):120 10.1016/j.arthro.2015.07.015 26413875

[8] Chahla J , Geeslin AG , Cinque ME , LaPrade RF Biomechanical proof for the existence of the anterolateral ligament. Clin Sports Med (2018) 37:33–40 10.1016/j.csm.2017.07.003 29173555

[9] Cianca J , John J , Pandit S , Chiou‐Tan FY Musculoskeletal ultrasound imaging of the recently described anterolateral ligament of the knee. Am J Phys Med Rehabil (2014) 93:186 10.1097/PHM.0000000000000070 24434891

[10] Claes S , Bartholomeeusen S , Bellemans J High prevalence of anterolateral ligament abnormalities in magnetic resonance images of anterior cruciate ligament‐injured knees. Acta Orthop Belg (2014) 80:45–49 24873084 24873084

[11] Claes S , Vereecke E , Maes M , Victor J , Verdonk P , Bellemans J Anatomy of the anterolateral ligament of the knee. J Anat (2013) 223:321–328 10.1111/joa.12087 PMC3791125 23906341

[12] Daggett M , Stephenson C , Dobson J , Whitaker A , Redler A , Monaco E , Wright B , Saithna A , Sonnery‐Cottet B Anatomic and histological study of the anterolateral aspect of the knee: a SANTI group investigation. Orthop. J Sports Med (2018) 6:2325967118799970 10.1177/2325967118799970 PMC6187433 30345320

[13] Dodds AL , Halewood C , Gupte CM , Williams A , Amis AA The anterolateral ligament: anatomy, length changes and association with the Segond fracture. Bone Jt J (2014) 96‐B:325–331 1:STN:280:DC%2BC2crgtFSisg%3D%3D. 10.1302/0301-620X.96B3.33033 24589786

[14] Ferretti A , Monaco E , Redler A , Argento G , De Carli A , Saithna A , Helito PVP , Helito CP High prevalence of anterolateral ligament abnormalities on MRI in knees with acute anterior cruciate ligament injuries: a case‐control series from the SANTI study group. Orthop J Sports Med (2019) 7:2325967119852916 10.1177/2325967119852916 PMC6593931 31263726

[15] Fulkerson JP , Gossling HR . Anatomy of the knee joint lateral retinaculum. Clin Orthop. 1980;(153):183–8. 7449213

[16] Getgood A , Brown C , Lording T , Amis A , Claes S , Geeslin A , Musahl V ALC Consensus Group The anterolateral complex of the knee: results from the international ALC consensus group meeting. Knee Surg Sports Traumatol Arthrosc (2019) 27(1):166–17617 10.1007/s00167-018-5072-6 30046994

[17] Hacker SP , Ignatius A , Dürselen L The influence of the test setup on knee joint kinematics ‐ a meta‐analysis of tibial rotation. J Biomech (2016) 49:2982–2988 10.1016/j.jbiomech.2016.07.025 27507625

[18] Helito CP , Helito PVP , Costa HP , Bordalo‐Rodrigues M , Pecora JR , Camanho GL , Demange MK MRI evaluation of the anterolateral ligament of the knee: assessment in routine 1.5‐T scans. Skelet Radiol (2014) 43:1421–1427 10.1007/s00256-014-1966-7 25085699

[19] Helito CP , Helito PVP , Leão RV , Demange MK , Bordalo‐Rodrigues M Anterolateral ligament abnormalities are associated with peripheral ligament and osseous injuries in acute ruptures of the anterior cruciate ligament. Knee Surg Sports Traumatol Arthrosc (2017) 25(4):1140–1148 10.1007/s00167-017-4498-6 28293698

[20] Hughston JC , Andrews JR , Cross MJ , Moschi A Classification of knee ligament instabilities. Part I. the medial compartment and cruciate ligaments. J Bone Joint Surg Am (1976) 58:159–172 1:STN:280:DyaE287kt1Gnuw%3D%3D. 10.2106/00004623-197658020-00001 1254619

[21] Hurley ET , Fried JW , Kingery MT , Strauss EJ , Alaia MJ Antero‐lateral ligament reconstruction improves knee stability alongside anterior cruciate ligament reconstruction. Knee Surg Sports Traumatol Arthrosc (2021) 29(3):764–771 10.1007/s00167-020-06002-8 32322949

[22] Inderhaug E , Stephen JM , Williams A , Amis AA Biomechanical comparison of anterolateral procedures combined with anterior cruciate ligament reconstruction. Am J Sports Med (2017) 45:347–354 10.1177/0363546516681555 28027653

[23] Johnson LL Lateral capsualr ligament complex: anatomical and surgical considerations. Am J Sports Med (1979) 7:156–160 1:STN:280:DyaE1M3ivFCktA%3D%3D. 10.1177/036354657900700302 464170

[24] Kittl C , El‐Daou H , Athwal KK , Gupte CM , Weiler A , Williams A , Amis AA The role of the anterolateral structures and the ACL in controlling laxity of the intact and ACL‐deficient knee. Am J Sports Med (2016) 44:345–354 10.1177/0363546515614312 26657572

[25] Kraeutler MJ , Welton KL , Chahla J , LaPrade RF , McCarty EC Current concepts of the anterolateral ligament of the knee: anatomy, biomechanics, and reconstruction. Am J Sports Med (2018) 46:1235–1242 10.1177/0363546517701920 28426251

[26] Kwak SD , Ahmad CS , Gardner TR , Grelsamer RP , Henry JH , Blankevoort L , Ateshian GA , Mow VC Hamstrings and iliotibial band forces affect knee kinematics and contact pattern. J Orthop Res (2000) 18(1):101–108 1:STN:280:DC%2BD3c7nvFShuw%3D%3D. 10.1002/jor.1100180115 10716285

[27] Musahl V , Herbst E , Burnham JM , Fu FH The anterolateral complex and anterolateral ligament of the knee. J Am Acad Orthop Surg (2018) 26:261–267 10.5435/JAAOS-D-16-00758 29517517

[28] Nitri M , Rasmussen MT , Williams BT , Moulton SG , Cruz RS , Dornan GJ , Goldsmith MT , LaPrade RF An in vitro robotic assessment of the anterolateral ligament, part 2: anterolateral ligament reconstruction combined with anterior cruciate ligament reconstruction. Am J Sports Med (2016) 44:593–601 10.1177/0363546515620183 26831632

[29] Oshima T , Nakase J , Numata H , Takata Y , Tsuchiya H Ultrasonography imaging of the anterolateral ligament using real‐time virtual sonography. Knee (2016) 23:198–202 10.1016/j.knee.2015.10.002 26810600

[30] Parsons EM , Gee AO , Spiekerman C , Cavanagh PR The biomechanical function of the anterolateral ligament of the knee. Am J Sports Med (2015) 43:669–674 10.1177/0363546514562751 PMC4708263 25556221

[31] Rahnemai‐Azar AA , Miller RM , Guenther D , Fu FH , Lesniak BP , Musahl V , Debski RE Structural properties of the anterolateral capsule and Iliotibial band of the knee. Am J Sports Med (2016) 44:892–897 10.1177/0363546515623500 26811306

[32] Rasmussen MT , Nitri M , Williams BT , Moulton SG , Cruz RS , Dornan GJ , Goldsmith MT , LaPrade RF An in vitro robotic assessment of the anterolateral ligament, part 1: secondary role of the anterolateral ligament in the setting of an anterior cruciate ligament injury. Am J Sports Med (2016) 44:585–592 10.1177/0363546515618387 26684663

[33] Sharifi M , Shirazi‐Adl A , Marouane H Sensitivity of the knee joint response, muscle forces and stability to variations in gait kinematics‐kinetics. J Biomech (2020) 99:109472 1:STN:280:DC%2BB3MjntlSgsw%3D%3D. 10.1016/j.jbiomech.2019.109472 31708244

[34] Slette EL , Mikula JD , Schon JM , Marchetti DC , Kheir MM , Turnbull TL , LaPrade RF Biomechanical results of lateral extra‐articular Tenodesis procedures of the knee: a systematic review. Arthroscopy (2016) 32(12):2592–2611 10.1016/j.arthro.2016.04.028 27324970

[35] Sobrado MF , Giglio PN , Bonadio MB , Helito PVP , Guimarães TM , Pécora JR , Gobbi RG , Helito CP Outcomes after isolated acute anterior cruciate ligament reconstruction are inferior in patients with an associated anterolateral ligament injury. Am J Sports Med (2020) 48:3177–3182 10.1177/0363546520956266 33017174

[36] Sonnery‐Cottet B , Lutz C , Daggett M , Dalmay F , Freychet B , Niglis L , Imbert P The involvement of the anterolateral ligament in rotational control of the knee. Am J Sports Med (2016) 44:1209–1214 10.1177/0363546515625282 26865395

[37] Spencer L , Burkhart TA , Tran MN , Rezansoff AJ , Deo S , Caterine S , Getgood AM Biomechanical analysis of simulated clinical testing and reconstruction of the anterolateral ligament of the knee. Am J Sports Med (2015) 43:2189–2197 10.1177/0363546515589166 26093007

[38] Taneja AK , Miranda FC , Braga CAP , Gill CM , Hartmann LGC , Santos DCB , Rosemberg LA MRI features of the anterolateral ligament of the knee. Skelet Radiol (2015) 44:403–410 10.1007/s00256-014-2052-x 25427785

[39] Trinchese GF , Oliva F , Maffulli N Minimally invasive anatomic reconstruction of the anterolateral ligament with ipsilateral gracilis tendon. Muscles Ligaments Tendons J (2017) 7:240–246 10.11138/mltj/2017.7.2.240 PMC5725172 29264334

[40] Van der Watt L , Khan M , Rothrauff BB , Ayeni OR , Musahl V , Getgood A , Peterson D The structure and function of the anterolateral ligament of the knee: a systematic review. Arthroscopy (2015) 31(3):569–82.e3 10.1016/j.arthro.2014.12.015 25744324

[41] Zappia M , Oliva F , Chianca V , Di Pietto F , Maffulli N Sonographic evaluation of the anterolateral ligament of the knee: a cadaveric study. J Knee Surg (2019) 32:532–535 10.1055/s-0038-1655763 29852517

